# Meningitis, Clinical Presentation of Tetanus

**DOI:** 10.1155/2015/372375

**Published:** 2015-02-18

**Authors:** Anna Moniuszko, Agata Zajkowska, Ewa Tumiel, Krzysztof Rutkowski, Piotr Czupryna, Sławomir Pancewicz, Ryszard Rutkowski, Agnieszka Zdrodowska, Joanna Zajkowska

**Affiliations:** ^1^Department of Infectious Diseases and Neuroinfections, Medical University of Bialystok, Zurawia 14, 15-540 Białystok, Poland; ^2^Medical University of Bialystok, Kilinskiego 1, 15-089 Białystok, Poland; ^3^Department of Allergy, Cambridge University Hospital, Hills Road, Cambridge CB2 0QQ, UK; ^4^Department of Respiratory Diagnostics and Bronchoscopy, Medical University of Bialystok, Waszyngtona 17, 15-274 Białystok, Poland

## Abstract

*Background*. Tetanus is an acute disease caused by a neurotoxin produced by *Clostridium tetani*. Tetanus immunization has been available since the late 1930s but sporadic cases still occur, usually in incompletely vaccinated or unvaccinated individuals. *Case Report*. An elderly previously vaccinated female contracted tetanus following foot injury. Clinically she presented with meningitis causing diagnostic and therapeutic delays. *Why Should Physician Be Aware of This?* Even in developed countries the differential diagnosis of meningitis, especially in the elderly, should include tetanus. Treatment in intensive care unit is required. General population might benefit from vaccine boosters and education on this potentially fatal disease.

## 1. Introduction

Tetanus is an acute disease caused by a neurotoxin produced by* Clostridium tetani*. Tetanus immunization has been available since the late 1930s but sporadic cases still occur, usually in incompletely vaccinated or unvaccinated individuals [1, 2]. Physicians' knowledge of this multifaceted condition is becoming scarce. It may cause diagnostic and therapeutic delays. We present a rare case of tetanus presenting as meningitis in a previously vaccinated patient.

## 2. Case Presentation

A 75-year-old Caucasian female, previously vaccinated against tetanus, was referred from Accident and Emergency Department (A&E) with suspicion of meningitis (severe headache, neck and back pain, trismus, photophobia, leg muscle spasms, and fever). Head CT was unremarkable. She was on warfarin (pulmonary embolism). ENT (ear, nose, and throat), neurology, and general medical consults were normal. Seven days previously she had stepped on a rusty nail and received tetanus toxoid in the general practitioner (GP) surgery one week after injury.

On admission she was conscious, dehydrated with neck stiffness, trismus, sublingual hemorrhages, and oliguria. Lower limbs and spinal muscles hypertonia with subsequent spasm and tachycardia with tachypnoea developed within 24 hours. Lumbar puncture was not performed. Creatinine and AST were raised. History and fast deterioration supported the diagnosis of tetanus. She was intubated in ICU and received rocuronium, baclofen, and 12 ampoules of tetanus serum (Igantet; 250 j.m./mL INSTITUTO GRIFOLS, S.A.). She remained intubated for 17 days. High CRP (64.5 mg/dL), anaemia (Hb 7.9 g/dL), and coagulopathy (INR 6.14; APTT 48.2 s; 32.7 s) developed. Coamoxiclav, metronidazole, antiulcer prophylaxis, and mucolytics were administered. After extubation she reported left leg pain/cramps. Decreased range of active and passive movements with swelling and ecchymosis of the left ankle were noted. The spasm resolved after rehabilitation outside ICU.

## 3. Discussion

Since 1940s the annual incidence of tetanus has reduced dramatically [3]. In England and Wales (1989/90–1995/96) it was 0.2 cases/million with the highest incidence in patients older than 64 years [4]. In Poland the incidence declined between 1966 (1.0/100.000 inhabitants) and 1999 (0.05/100.000). Nevertheless around 40 new cases are reported annually. In 2011 all of 14 cases reported (35% mortality) were in patients older than 50 (incidence 0.036/100.000). Only 4 persons had been vaccinated [5].

Generalized tetanus is the most common. Trismus (lockjaw) is usually the first symptom. Autonomic overreactivity causes irritability, restlessness, sweating, and tachycardia. Profuse sweating, cardiac arrhythmias, labile hypertension or hypotension, and fever may develop. In generalized tetanus tonic contractions of skeletal muscles and intermittent intense muscular spasms are common. They are responsible for neck stiffness, opisthotonus,* risus sardonicus*,* board-like* rigid abdomen, periods of apnea, and/or upper airway obstruction due to contraction of the thoracic/glottal/pharyngeal muscles and dysphagia [6, 7]. Severe neck stiffness, fever, and headache in our patient led to suspicion of meningitis. Photophobia, hyperacusis, anxiety, and increased muscle tone may also be present in meningitis. This may cause diagnostic and therapeutic delays. Fortunately a detailed clinical history revealed a preceding contaminated wound.

Differential diagnosis of tetanus should include drug-induced dystonias, trismus due to dental infection, strychnine poisoning, malignant neuroleptic syndrome, or stiff-person syndrome [8]. Our patient had symptoms suggesting meningitis.

The diagnosis does not require laboratory confirmation [7]. There were no specific findings in our case.

Tetanus toxin binds irreversibly to tissues; only unbound toxin can be neutralized by passive immunization. It is considered standard treatment and improves survival [9, 10]. Our patient presented to GP days after injury and received the toxoid too late.

Despite effective vaccination, sporadic cases of this rare disease are reported. It should be considered in the differential diagnosis of meningitis, especially in the elderly. Public health measures include education on management of contaminated wounds and 10-yearly vaccination boosters [11].
